# The Influence of Surface Topography on the Weak Ground Shaking in Kathmandu Valley during the 2015 Gorkha Earthquake, Nepal

**DOI:** 10.3390/s20030678

**Published:** 2020-01-26

**Authors:** Mark van der Meijde, Md Ashrafuzzaman, Norman Kerle, Saad Khan, Harald van der Werff

**Affiliations:** 1 Department of Earth Systems Analysis, Faculty of Geo-Information Science and Earth Observation (ITC), University of Twente, 7500 AE Enschede, The Netherlands; ashrafrhd27@gmail.com (M.A.); n.kerle@utwente.nl (N.K.); saadkhan@bkuc.edu.pk (S.K.); harald.vanderwerff@utwente.nl (H.v.d.W.); 2 Roads and Highways Department, Government of the People’s Republic of Bangladesh, Sarak Bhaban, Tejgaon, Dhaka 1208, Bangladesh; 3 Department of Geology, Bacha Khan University Charsadda, Charsadda 24420, Pakistan

**Keywords:** earthquake, Nepal, amplification, modelling, digital elevation model

## Abstract

It remains elusive why there was only weak and limited ground shaking in Kathmandu valley during the 25 April 2015 Mw 7.8 Gorkha, Nepal, earthquake. Our spectral element numerical simulations show that, during this earthquake, surface topography restricted the propagation of seismic energy into the valley. The mountains diverted the incoming seismic wave mostly to the eastern and western margins of the valley. As a result, we find de-amplification of peak ground displacement in most of the valley interior. Modeling of alternative earthquake scenarios of the same magnitude occurring at different locations shows that these will affect the Kathmandu valley much more strongly, up to 2–3 times more, than the 2015 Gorkha earthquake did. This indicates that surface topography contributed to the reduced seismic shaking for this specific earthquake and lessened the earthquake impact within the valley.

## 1. Introduction

Considering the magnitude and location of the 2015 Mw 7.8 Gorkha, Nepal, earthquake, it had much less impact in Kathmandu valley than expected [1,2,3,4], with only about one-third of the estimated ground shaking [4]. Macroseismic intensities in Kathmandu valley were of the order of 6–7 on the European Macroseismic Scale (EMS) [5], as compared to a predicted 8 EMS [2]. Despite 9000 fatalities and 23,000 people injured across Nepal, mainly caused by the collapse of buildings [6,7], the impact in Kathmandu valley was 1700 deaths and 11,000 people injured [7], much less than the predicted 100,000 fatalities and 200,000 injured for a comparable seismic event [8]. This substantially lower impact was a result of relatively weak shaking and low-frequency content generated by this major earthquake [1,2,9,10] and specific conditions along the seismic propagation path and in the bowl-shaped and sediment filled Kathmandu valley. As a result, the structural damage of buildings was less pronounced in the valley interior than along its margins [11].

**Figure 1:**
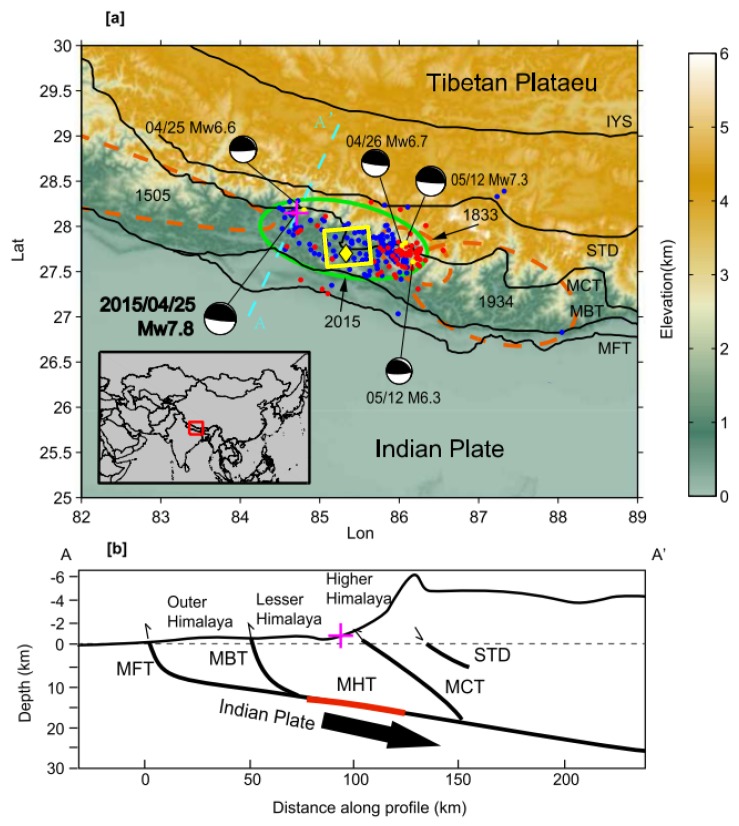
Seismo-tectonic setting of the Himalayan region (modified with permission from [27]) (**a**) tectonic setting of the Himalayan Region with topography. MFT = Main Frontal Thrust, MBT = Main Boundary Thrust, MCT = Main Central Thrust, STD = Southern Tibetan Detachment System, IYS = Indus–Yarlung Suture. The purple cross is the epicentre of the main shock and yellow dots are large aftershocks. Focal mechanisms are shown by black and white beach balls. The blue and red dots are aftershocks (Mw > 3.5 that occurred between the mainshock (25 April 2015) and the largest aftershock Mw 7.3 (12 May 2015), and after the largest aftershock till 30 May 2015, respectively. The brown ellipses show rupture areas of the past 1934 (Mw 8.1), the 1833 (Mw 7.6) and the 1505 (Mw 8.2) earthquakes. The green ellipse shows the approximate fault plane of the 2015 Mw 7.8 earthquake. The yellow square is the study area on which this research is performed. (**b**) cross section along AA’ in (**a**) that shows the approximate location of slip of Mw 7.8 earthquake with epicenter location (purple cross) and approximate fault rupture (red line). MHT = Main Himalayan Thrust.

To explain the limited seismic shaking inside Kathmandu valley, an identification of the ground motion distribution pattern is crucial. Several studies have characterized different aspects of the source mechanism and site effects during this event. These include (i) the confinement of the rupture to a shallow zone of the Main Himalayan Thrust system [12,13,14,15], (ii) the characteristics of the source with low-frequency radiation of seismic energy (0.05–2.0 Hz) and a relatively smooth onset [9,12,13,14,15,16,17,18,19] in relation to the type of buildings in Kathmandu valley [20], and (iii) the role of valley sediment deposits in amplifying seismic ground motion [19,21,22,23]. Many studies have focused on the rupture process, and related tectonic constraints [9,12,13,14,15,17,18,24,25], and the sediment response in the bowl-shaped Kathmandu valley [19,21,22,23]. These studies could partly explain the limited fatality rate and physical destruction, and to some extent the damage distribution pattern within Kathmandu valley. There are, however, still questions remaining that cannot be resolved with our present knowledge of the event. To be able to explain the spatial distribution of the impact of a seismic event, the understanding of the interaction of seismic waves with (sub)surface structure along the path from source to a site is critical. After initiation at a shallow depth (around 15 km, see reference above) in the Gorkha region, 80 km northwest of Kathmandu city, the earthquake rupture propagated eastward towards Kathmandu valley. The maximum energy release was 22 km north of Kathmandu city at relatively shallow depth [9,12,13,25]. Because of the intense surface topography between Kathmandu valley and the location of maximum seismic energy release (Figure 1), a seismic wave is expected to show constructive or destructive interference and subsequent amplification or de-amplification [26], respectively, which could significantly change the intensity of ground motion inside the valley.

Here, we analyze the role of surface topography on ground motion distribution through a full elastic finite element seismic wave simulation. To fully appreciate the effect of directional propagation and interaction with topography, we have selected five potential fault source locations along the fault, including the centroid moment tensor (CMT) location (based on the United States Geological Survey (USGS) fault plane solution, [25]). We modeled the interaction of the seismic wavefield with a high-resolution Earth surface model of the valley and surrounding area for each of these locations. We determined for each fault source location the effect of topography on seismic shaking in Kathmandu valley at bedrock level. This is at the bottom of the sediments inside the valley, and the interaction with sediment deposits inside the valley is not included to avoid having multiple effects overprinting each other. Defocusing of seismic energy, while propagating towards Kathmandu city, as well as de-amplification of seismic waves inside the valley by surface topography, gives insight into the possible role of topography in the observed weak ground shaking.

## 2. Data and Methods

**Figure 2:**
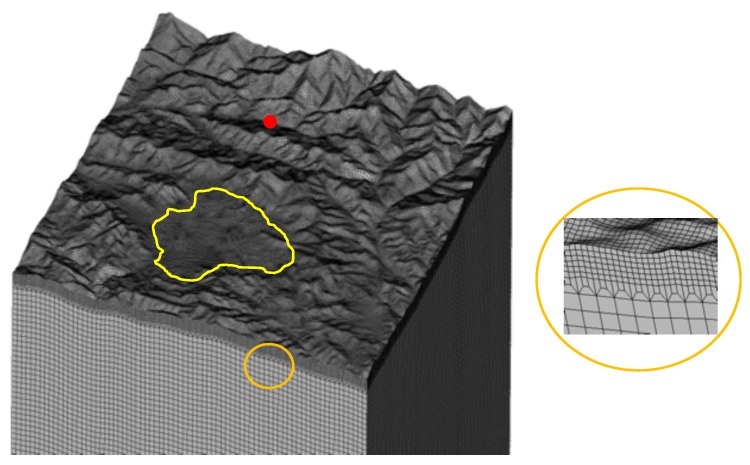
Spectral Element Mesh. (**Left**) The mesh for Kathmandu valley and surrounding areas. (**Right**) The enlarged version of near-surface mesh showing the realistic surface topography and triplication layer that has been applied. For sizes of the total area, cells and resolution of topography, see details in text and Table 1.

We used the spectral element method (SEM) [28,29] to develop a 3D hexahedral mesh (Figure 2) that incorporates realistic satellite-based surface topography of Kathmandu valley and its surrounding areas. The size of the 3D model is 57.9 km, 56.5 km, and 57.0 km in length, width, and height, respectively (see Table 1). We also developed a flat model (without surface topography) to compare to the realistic topography model.

### 2.1. Satellite Based Topography

The Shuttle Radar Topography Mission (SRTM) digital elevation model (DEM) was used to represent realistic surface topography (Figure 2). We chose the SRTM DEM because it is more consistent with elevation and morphometric properties compared to other satellite-based DEM [30]. It is vital to select the appropriate resolution of the DEM for 3D simulations of seismic response. Using a high-resolution DEM is accurate but generates a high number of spectral elements that make SEM computationally very expensive. On the other hand, a DEM resolution coarser than 540 m in spectral element simulations produces less accurate results [26]. Based on the available processing capacity, we used an SRTM DEM 9 arc-second grid spacing for incorporating realistic surface topography into an SEM 270 m hexahedral mesh.

### 2.2. Model Construction

We constructed the mesh by using CUBIT in conjunction with GeoCubit [31,32], see for all mesh details also Table 1. We developed a mesh of homogeneous half-space type (Figure 2), which was a single block consisting of elastic materials.

The element size (d) was determined by [33],

(1) $$d=\frac{N}{5}V_{min}T_{0},$$

where $V_{min}$ is the minimum seismic wave speed, and $T_{0}$ is the shortest period. A polynomial degree N=4 was used to sample the wavefield [26]. To ensure a stable time scheme for the spectral-element simulation, the selection of time-step Δt during the simulation of the seismic wave through the mesh was restricted by [33]

(2) Δt≤Cmin_Ω(Δx/V).

Here, *C* is the Courant stability number that was empirically derived to be around 0.3 [34], Ω is the model volume, Δx is the distance between two Gauss–Lobatto–Legendre (GLL) nodes, and *V* is the P-wave speed. An acceptable level of geometrical distortion of elements with maximum skewness of <0.8 [33] was considered.

We designed the mesh to resolve seismic waves with the shortest period of 0.57 s (up to 1.75 Hz frequency). The average GLL distance in the horizontal direction at the surface was 60–61 m, which is small enough to be resolved with 9 arc seconds grid spacing DEM data [26]. The mesh properties and simulation parameters are shown in Table 1.

Each element was defined with material properties, including the velocity of the P-wave ($V_{p}$) and S-wave ($V_{s}$), the density of the material, and attenuation.

Uniform velocities of P-wave (Vp=5850 m/s) and S-wave (Vs=3370 m/s) were used for simulation. The Vp and Vs were an average of the (upper)crustal velocities used in several studies on Nepal or for the Himalayan region [12,35,36,37]. These models all have a coarse resolution (over 100 km) or are point observations at individual locations or along profiles. We have therefore adopted an average value for our, relatively small, region due to a lack of more detailed specific knowledge of velocities for each specific location in our model.

Because of lack of information, the density value ρ was set at 3230 ${\mathrm{kg}/m}^{3}$, based on the empirical formula [33]

(3) $$\rho =V_{p}/3+1280.$$

The attenuation in bedrock, at the bottom of the sediments, was assumed to be zero. As a consequence, the only loss of energy is due to geometrical spreading. Since both the flat and the topographic model have the same properties, this will not influence the amplification calculations since we take the ratio between the two models that have experienced equal attenuation (if there was any).

### 2.3. Earthquake Simulation

The constructed mesh was exported into a SPECFEM3D Cartesian file format. SPECFEM3D is an open-source code developed for simulating acoustic, elastic, coupled acoustic/elastic, poroelastic, or seismic wave propagation based on the principles of SEM [34]. The source used for simulation of the 2015 Gorkha earthquake was the global centroid moment tensor (CMT) solution [38]. The CMT is the center of earthquake energy distribution and the location with dominant moment release, defined by six moment tensor components and centroid coordinates, depth, and centroid time [34,38].

Note that our study area (approximately 58 km × 57 km) is 11 times smaller than the whole fault plane (approximately 220 km × 165 km) [25] of the earthquake. Since we use a very high resolution model, modelling the whole fault would be extremely computationally expensive. Furthermore, we are interested in the directional effect of the interaction with topography, particularly for the high-intensity waves originating from the fault segment with highest energy release very close to Kathmandu. We have therefore focused on the CMT location as the main source of energy for the earthquake and as a representation of the whole fault plane. It also allowed exploring the effect of potential alternative CMT locations, as alternative earthquake scenarios, at other places along the fault to test the directional effect of seismic wave propagation in relation to geometrical effects of the topographic (de-)amplification.

### 2.4. Simulation Output

After the simulation, the peak ground displacement (PGD) and synthetic seismogram were generated. The PGD parameter was used for characterizing ground motion because it is mostly associated with lower frequency components of seismic motion [39], and in-line with the dominant low-frequency content of the earthquake, and SEM can simulate such low-frequency displacements [40]. We used the PGD amplification factor [41] to quantify differences in PGD between the two models. The PGD amplification map was produced by using the equation [26,41]

(4) $$SAF=\frac{PGD_{A}-PGD_{B}}{PGD_{B}}*100\%,$$

where SAF = seismic amplification factor, $PGD_{A}$ and $PGD_{B}$ are peak ground displacement for models with realistic topography and with flat topography, respectively.

We compared the synthetic peak ground acceleration (PGA) generated by our model with observed PGA recordings. Since we only considered surface topography (and no sediments), we compared the synthetic PGA with recorded data from the Kirtipur Municipality Office accelerometer station (KTP), which is the only seismic station in the region located on bedrock [23]. Before comparison, the synthetics were deconvolved with the CMT source time function with proper half duration (since a zero half duration was used in the modeling [34]), and the instrument response (Mitutoyo JEP-6A3-2) was removed from the seismic observations. Both were re-sampled to 10 Hz and filtered between 5–20 s since there was a predominantly low frequency (0.05–0.2 Hz) emission of seismic energy near Kathmandu valley [9,15,16,42].

### 2.5. Evaluation of Directional Effects

Since the occurrence of de-amplification strongly depends on the location, we evaluated alternative earthquake scenarios to explore the impact of topography along the source-site propagation path. We selected four alternative locations, based on the United States Geological Survey (USGS) finite fault model [25]. Locations P1 to P4 correspond to centers of the four sub-faults that had the most significant release of seismic moment along the main rupture after the primary CMT location used so far. Simulation results are based on the same CMT characteristics for each of the locations to make the results comparable.

## 3. Results

### 3.1. Model Results

**Figure 3:**
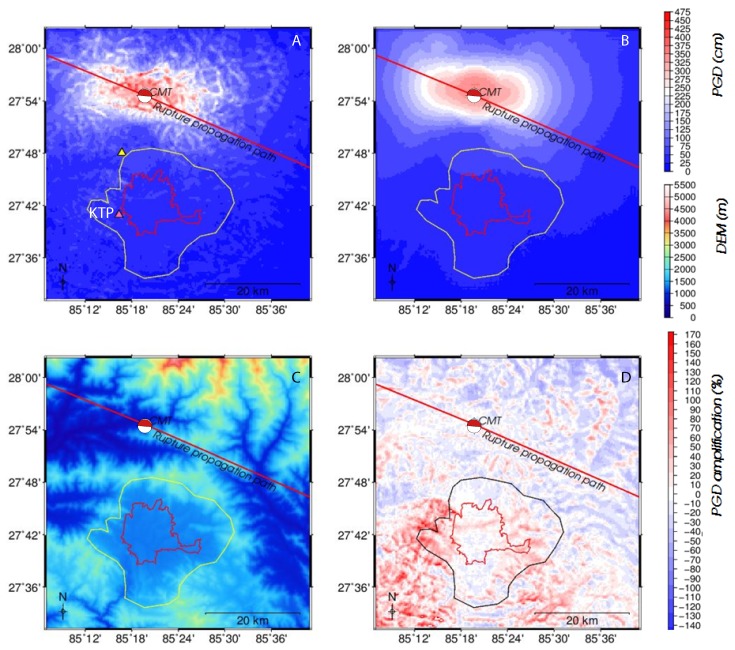
Surface topography and peak ground displacement (PGD) model for the 25 April 2015 Mw 7.8 Nepal Earthquake. (**A**) absolute PGD for a model with surface topography. The recorded data at Kiritipur municipality office accelerometer station (KTP), located on bedrock, is compared with the synthetic seismogram. (**B**) absolute PGD for a model without realistic surface topography. (**C**) Shuttle Radar Topography Mission (SRTM) digital elevation model of the study area; (**D**) PGD amplification map (in %). The rupture propagation path (red line) indicates the path of dominant release of seismic energy.

The simulation for the model with topography included (Figure 3A) shows an irregular pattern of ground motion, a result of reflection and scattering of seismic energy by surface topography (Figure 3C). In contrast, the flat model generated a smooth distribution of PGD (Figure 3B). In the realistic topography model, the valley interior experiences less PGD compared to the flat surface model, and the sides of the valley are having a higher PGD. Due to the loss of energy of the seismic waves, the amplitudes decrease very fast, and the comparison for lower amplitudes is not very easy to derive from the PGD Figure 3A,B. The contrast between the topographic and the flat model, for Kathmandu valley, can be best seen in Figure 3D, where the amplification factor is shown based on the division of the topography model by the flat model. This provides insight into the scattering effect of mountains on seismic energy propagation. Around the source region, where the waves are most vertically coming up from the source, there is very little to no interaction of the seismic waves with the topography, and the amplification factor shows a scattered pattern around zero amplification. In between the CMT source and the valley are two mountain ranges oriented almost perfectly perpendicular to the propagation direction (Figure 2 and Figure 3C). Seismic waves travelling from the source to Kathmandu valley will interact with these mountains, and the topography will scatter and influence the direction of propagation of the seismic waves. Most parts of the inner Kathmandu valley have zero or negative PGD amplification factors (Figure 3D), indicative of de-amplification of seismic energy. The mountains scattered and deflected seismic energy and directed most energy to the eastern and western margins of the valley (Figure 3D). As a result, a shadow zone of seismic energy occurred in the inner part of Kathmandu valley. At the same time, the de-focused energy produced up to a three times higher amplification along the Kathmandu valley periphery (Figure 3D), which explains why damages were more pronounced at the edges of the valley than in its center.

### 3.2. Model Validation

**Figure 4:**
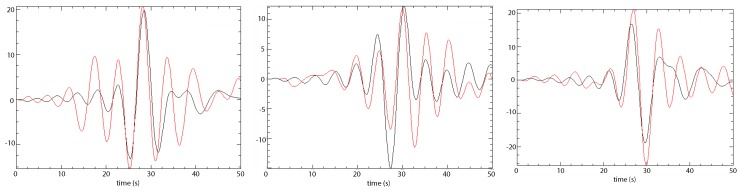
Comparison between the observed (black line) and synthetic (red line) time histories of peak ground acceleration (PGA) (cm/s${}^{2}$). The three components N, E and Z are presented.

The validity of the simulations was tested through a comparison with a seismic observation at a seismometer at the western side of Kathmandu valley (Figure 4). The ground motions of the 2015 Gorkha earthquake recorded throughout the Kathmandu Valley were studied in Takai et al. [23]. Four station locations were available throughout the Kathmandu Valley. These were: KTP, TVU (Central Department of Geology, Tribhuvan University, Kirtipur), PTN (Pulchowk Campus, Institute of Engineering, Tribhuvan University, Patan), and THM (University Grants Commission Office, Sanothimi, Bhaktapur), which are distributed from west to east. The KTP-station was established on a rocky soil, representative of the motion at the bedrock level. The observations at KTP are, therefore, comparable to the modeled seismic signal. The remaining three sites were established on sediments and, since these are not modelled, they cannot be used for validation. Comparison of the synthetic seismograms with real data (Figure 4) shows that both the timing and amplitude of the main seismic waves are consistent in timing and comparable in amplitude. The small misfits might be due to the uniform velocities applied in our model and simplification of (near) subsurface structure in the model.

### 3.3. Evaluation of Directional Effects

**Figure 5:**
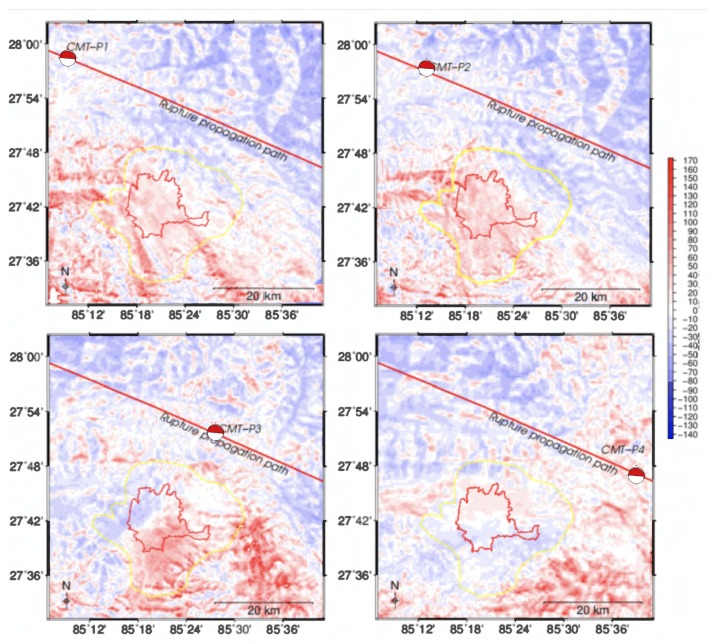
PGD amplification for scenarios with centroid moment tensor (CMT) at four different locations (P1 to P4) along the fault-rupture line. Subfigures show the expected amplification outcomes for maximum seismic energy release at points P1, P2, P3, and P4, as indicated in the figure.

The results obtained for locations P1 to P4 show different amplification results (Figure 5). If the maximum energy had been released approximately 20 km (P1 scenario) and 10 km (P2 scenario) northwest from the actual position, the topographic shielding would have been ineffective. Almost the entire densely populated area would have received a high amplification of 30% to 120% (Figure 5). The amplification in Kathmandu valley would have been much higher than the overall de-amplification modelled for the actual scenario (based on the primary CMT location). In the P3 scenario, where the source is 15 km southeast from the actual position, amplification would be limited to the southern part of the valley, but with amplitudes for the amplification exceeding 100% (Figure 5). The northwestern part of the valley shows de-amplification, like the original scenario. Finally, if the earthquake center had been 30 km southeast from its actual position (P4 scenario), the shielding effect would again have prevented significant amplification in the entire valley, except for some areas in the northern part of the valley where amplification in the range of 20–40% is modelled (Figure 5).

## 4. Discussion

It should be emphasized that we study here only the topographic effects and that this study is not meant to be a full finite element model of the complete finite fault model. To highlight the topographic effect, we have reduced the complexity of a potential full realistic model to a simpler model that shows explicitly the topographic directional effect for this specific earthquake. It is evident from the models that topography has played a role in the propagation of seismic waves through the region. However, we have also simplified a few components to isolate the effect of topography. We have, for example, not considered the sediment effect in the valley. Our model only shows the expected motion at the bedrock, the bottom of the sediments. Adding the sediments would have been an option but was not done for two reasons. The first was that no sediment information was available at the resolution of our model (270 m). The second reason was that sediments would have overprinted the topography effect to the extent that the topography effect would not be uniquely visible anymore. We have also simplified the source mechanism and used point sources for all scenarios. Realistically, for a full analysis, a complete fault model is required. We have tested this to see its effect, but the different solutions, as shown in Figure 3 and Figure 5, will partly overprint each other, resulting in either enhancement or destruction of amplitudes, thereby masking the effect of specific directions. The solution nearby, with the most substantial energy release (with a displacement twice as high as the displacement at any of the other selected locations [25]) has a strong influence on the final results, but the other sub-fault effects mask part of the specific topographic effects. To fully appreciate the spatial and directional dependence of the (de-)amplification with respect to the topography, we have hence shown solutions of the primary CMT location and the same source mechanism at the four strongest individual sub-fault locations along the main rupture of the USGS finite fault model [25]. A final, and complete, study should use information from the source (like [9,16,17,24,25] and others), the path (like this study), the sediments (like [19] and others), and the specific buildings, particularly height and building style (like [20] and others) for resonance studies. Such a combined study is the only option to get an appreciation of the full impact of the earthquake. Thus far, it is too complicated to merge all these in a single analysis. It is unclear what the total effect on Kathmandu valley is of the low-frequency content and slow onset of the earthquake on Kathmandu valley. The resulting sediment effect is partly a result of the subsurface conditions, but also the frequency content of the incoming wave. It cannot be derived from literature at this moment what the exact contribution is of the specific subsurface conditions and to what extent the low-frequency content; in combination with the specific subsurface conditions, this has resulted in lower impact on the Kathmandu valley. All these components have their own contributions, and each of the elements will add a piece to the puzzle of why Kathmandu has escaped severe damage and a much higher mortality rate.

## 5. Conclusions

Despite the proximity to the densely populated Kathmandu valley, the April 2015 Gorkha earthquake was less damaging than predicted. However, the impact could have been worse if the earthquake had released its main energy in a slightly different location. Our modelling of the seismic wave propagation through the intense topography north of Kathmandu valley showed that the topographic effect on the seismic wave propagation led to a de-amplification of the seismic energy potentially reaching the valley. We evaluated different potential earthquake scenarios, by shifting the main source along the main rupture. The models show that, for maximum energy at alternative locations, some locations show a similar de-amplification for (parts) of the Kathmandu valley. Other locations, particularly located NW of the present CMT location, show very strong amplification effects, exceeding 100% amplification in some scenarios. We have shown that the relatively weak shaking can be, partly, contributed to regional topography and has a contribution to the observed weak ground shaking for Kathmandu valley.

## Figures and Tables

**Table 1:** Mesh properties and simulation paramters.

Model Parameters and Simulation Process	Mesh (with Topo)	Mesh (without Topo)
Mesh dimension (km)	56.5 * 57.9 * 57	56.5 * 57.9 * 57
Total number of elements (million)	0.855	0.842
Average GLL distance at surface (m)	61.25	60.5
Number of grid points (million)	56.3	55.2
Number of degrees of freedom (million)	168.9	165.6
Mesh slices (number of processors)	16	16
Simulation time (seconds)	90	90
Number of time steps	300,000	300,000
Step (seconds)	0.0003	0.0003
